# Inactivation of the lysine binding sites of human plasminogen (hPg) reveals novel structural requirements for the tight hPg conformation, M-protein binding, and rapid activation

**DOI:** 10.3389/fmolb.2023.1166155

**Published:** 2023-04-04

**Authors:** Yetunde A. Ayinuola, Francis J. Castellino

**Affiliations:** ^1^ W. M. Keck Center for Transgene Research, Notre Dame, IN, United States; ^2^ Department of Chemistry and Biochemistry, University of Notre Dame, Notre Dame, IN, United States

**Keywords:** plasminogen, plasminogen activation, streptococcal M-protein, streptokinase, protein mutagenesis, bacterial M-protein, *Streptococcus pyogenes*

## Abstract

Accelerated activation of the human plasminogen zymogen (hPg) to two-chain active plasmin (hPm) is achieved following conformational changes induced by ligand-binding at the lysine-binding sites (LBSs) in four of the five hPg kringle domains. In this manner, pattern D skin-trophic strains of Group A streptococci (GAS), through the expression of surface plasminogen-binding M-protein (PAM), immobilize surface hPg, thereby enabling rapid hPg activation by GAS-secreted streptokinase (SK). Consequently, GAS enhances virulence by digesting extracellular and tight cellular junctional barriers using hPm activity. Many studies have demonstrated the singular importance of the kringle-2 domain of hPg (K2_hPg_) to PAM-binding using hPg fragments. Recently, we showed, using full-length hPg, that K2_hPg_ is critical for PAM binding. However, these studies did not eliminate any modulatory effects of the non-K2_hPg_ LBS on this interaction. Moreover, we sought to establish the significance of the intramolecular interaction between Asp^219^ of the LBS of K2_hPg_ and its serine protease domain binding partner, Lys^708^, to conformational changes in hPg. In the current study, selective inactivation of the LBS of K1_hPg_, K4_hPg_, and K5_hPg_ revealed that the LBS of these kringle domains are dispensable for hPg binding to PAM. However, the attendant conformational change upon inactivation of K4_hPg_ LBS increased the affinity of hPg for PAM by an order of magnitude. This finding suggests that the native hPg conformation encloses PAM-binding exosites or sterically hinders access to K2_hPg_. While simultaneous inactivation of the LBS of K1_hPg_, K4_hPg_, and K5_hPg_ inhibited hPg/SK association alongside hPg activation, the replacement of Lys^708^ generated a slight conformational change that optimally accelerated hPg activation. Thus, we accentuate disparate functions of hPg LBS and conclude, using intact proteins, that K2_hPg_ plays a central role in regulating hPg activation.

## Introduction

Activation of the single-chain human plasminogen (hPg) following the scission of its Arg^561^-Val^562^ bond to produce the two-chain proteolytically active plasmin (hPm) is an essential step in the process leading to the dissolution of the fibrin blood clot (Urano et al., 2018). In addition to this principal function, hPm exhibits a broad substrate specificity catalyzing the degradation/activation of extracellular matrix (ECM) proteins, such as collagen, fibronectin, matrix metalloproteinase, and collagenase (Wong et al., 1992; Andreasen et al., 2000; Wu et al., 2000; Miles et al., 2005). Moreover, many cell types, including blood, immune, and bacterial cells, are endowed with hPg receptors, enabling them to co-opt the proteolytic activity of hPm for their diverse functions (Bhattacharya et al., 2012; Plow et al., 2012). Consequently, hPm is thought to play a significant role in regulating ECM integrity, cell migration, and the outcome of bacterial infection.

hPm formation/activity is regulated by at least three mechanisms. One such regulatory mechanism is intrinsic to the hPg molecule, wherein this protein exists in a closed, slowly activatable conformation. hPg is a 791 amino acid residue, single-chain glycoprotein that exists in at least seven distinct domains. Residues Glu^1^-Lys^77^ serve the function of an activation peptide (AP) in the native hPg (Glu^1^-hPg). The AP domain is followed by five consecutive kringle domains (K1_hPg_–K5_hPg_), each of ∼80 residues, characterized by triple disulfide bonds and lysine binding sites (LBS). These LBS are used for ligand binding and are typically constituted by anionic, cationic, and hydrophobic centers that interact and coordinate with amino, carboxylate, and methylene groups of lysine (analogs and isosteres), respectively, in the order of K1_hPg_ > K4_hPg_ > K5_hPg_ > K2_hPg_ in decreasing binding affinity (McCance et al., 1994; McCance and Castellino, 1995). K3_hPg_ LBS is inactive and has no measurable affinity for lysine due to the presence of Lys^311^ in place of Glu/Asp of the anionic center (Marti et al., 1994). The seventh and last domain, the serine protease (SP) domain, comprises residues Val^562^-Asn^791^, together with the AP domain, participate in intramolecular interactions that occupy the LBS of K2_hPg_, K4_hPg_, and K5_hPg_, retaining hPg in an activation-resistant closed conformation (Urano et al., 1988; Law et al., 2012). Disruption of these intramolecular interactions by limited proteolysis of Glu^1^-hPg through the action of preformed hPm that cleaves the AP domain to generate Lys^78^-Asn^791^ (Lys^78^-hPg) opens the hPg protein, making it more readily activatable (Markus et al., 1976; Urano et al., 1987a). Similarly, the binding of hPg to fibrin clot and cell surface receptors liberates the AP domain from the LBS of hPg, thereby relaxing its conformation (Miles and Parmer, 2013).

PAM, plasminogen-binding group A *Streptococcus* (GAS) M-protein, is a hPg receptor found on the surface of pattern D skin-trophic strains of *S. pyogenes* (Berge and Sjobring, 1993; Wistedt et al., 1995). GAS is a strict human pathogen responsible for various diseases, ranging from mild throat and skin infections to severe and life-threatening illnesses such as necrotizing fasciitis and toxic shock syndrome (Carapetis et al., 2005). We have demonstrated, with the aid of through-space lysine isosteres of PAM (Rios-Steiner et al., 2001; Yuan et al., 2019; Qiu et al., 2020), that the acquisition of hPg and its conversion to hPm by the endogenous GAS hPg activator, streptokinase (SK), assists GAS in the degradation of barriers to its dissemination (Vu et al., 2021). Importantly, we showed that PAM binding to K2_hPg_ displaces the intramolecular interactions maintained by this domain, giving rise to a conformational switch that expedites streptokinase-mediated activation of hPg (Bhattacharya et al., 2014; Ayinuola et al., 2021). Likewise, the replacement of Asp^219^ of the anionic center of K2_hPg_ LBS, (Ayinuola et al., 2021) while abolishing PAM-binding, produces a hPg with a relaxed conformation having a rapid and optimal activation potential (Ayinuola et al., 2021).

K2_hPg_ is the weakest lysine-binding domain of hPg, and structural studies have shown that it does not interact with the AP domain that is classically known to maintain hPg in the closed conformation. Instead, K2_hPg_ interacts with the SP domain, wherein Asp^219^ forms a salt bridge with Lys^708^ (Law et al., 2012). In the current study, we further investigated the significance of the interaction between Asp^219^ and Lys^708^, which is yet to be fully established, to the conformational and activation properties of hPg. Moreover, it is not yet known if the LBS of K1_hPg_, K4_hPg_, and K5_hPg_, in the intact hPg molecule, is important in enhancing or stabilizing the interaction between PAM and hPg. Herein, using site-directed mutagenesis of critical LBS residues, we investigated the influence of these residues on hPg conformation, activatability, and PAM-binding. These mutations also allowed us to highlight the residues of hPg involved in its interaction with SK and hPg activators, *viz.*, urinary-type plasminogen activator (uPA) and tissue-type plasminogen activator (tPA).

## Materials and methods

### Construction, expression, and purification of recombinant proteins

#### Plasminogen

The cDNA encoding wild-type (WT) Glu^1^-hPg was inserted into multiple cloning sites of the *Drosophila S2* parent expression plasmid, pMT-PURO, to generate hPg WT-pMT-PURO (Nilsen et al., 1999). A detailed description of the construction of the pMT-PURO plasmid has been provided elsewhere (Iwaki and Castellino, 2008). Five other variants of Glu^1^-hPg, *viz.*, hPg [D^139^N], hPg [D^413^N], hPg [D^518^N], hPg [D^139,413,518^N], and hPg [K^708^A], were generated via primer-directed mutagenesis PCR using the primers listed in Table 1. As an example, to construct the plasmid encoding hPg [D^139^N], two PCR fragments were generated using hPg-pMT-PURO as a template. Primers K1f and K1_LBS_r generated fragment-1, while primers K1_LBS_f and K1r generated fragment-2. These fragments were subsequently joined in an overlapping PCR using primers K1f and K1r. All other variants, except for hPg [D^139,413,518^N]-pMT-Puro, where hPg [D^413^N]-pMT-PURO was used as the template, were similarly constructed using primers listed in Table 1. For each construct, the final PCR fragment was inserted into the pCR Blunt II TOPO vector (Invitrogen) and transformed into TOP10 *Escherichia coli* cells for propagation. After verifying the accuracy of the sequence, particularly the presence of the desired mutation by Sanger DNA sequencing, the hPg fragments in the PCR TOPO vectors were digested using appropriate restriction enzymes and ligated into WT-hPg-pMT-PURO, which was similarly digested. In the case of hPg [D^139,413,518^N], the digested fragment was ligated into hPg [D^139^N]-pMT-PURO. The construction of the plasmid coding for hPg [D^219^N] was previously reported (Ayinuola et al., 2021).

**TABLE 1:** Primers for construction of hPg variants.

**Construct**	**Primer**	**Sequence^a^**
hPg [D^139^N]	K1f	5′-CTTTGTTGGCCTCTCGCTCG-3′
	K1_LBS_r	5′-CCTGCGGattGTTGTCTGGATTC-3′
	K1_LBS_f	5′-GAATCCAGACAACaatCCGCAGG-3′
	K1r	5′-GTGCACTCCAGTGCTGACAGG-3′
hPg [D^413^N]	K4_LBS_r	5′-CAGGGGCCTTTattGGCATCTGGATTCC-3′
	K4_LBS_f	5′-GGAATCCAGATGCCaatAAAGGCCCCTGGTG-3
hPg [D^518^N]	K5_LBS_r	5′-CCAGGGACCACCTACattACCATCAGGGTTACG-3′
hPg [D^139,413,518^N]	K5_LBS_f	5′-CGTAACCCTGATGGTaatGTAGGTGGTCCCTGG-3
hPg [K^708^A]	Pg_708_r	5′-CGATTGCACACTgcaTTCTCAATCACAGG-3′
	Pg_708_f	5′-CCTGTGATTGAGAATgcaGTGTGCAATCG-3′
hPg [D^413^N]	Pf^b^	5′-CCAGGCCTGGGACTCTCAGAG -3′
hPg [D^518^N]		
hPg [D^139,413,518^N]	Pr^b^	5′-TAGAAGGCACAGTCGAGG-3′
hPg [K^708^A]		

^a^Nucleotides written in lower case indicate mutations in the WT-Glu^1^hPg sequence.

^b^ Primers Pf and Pr were used as external primers and in overlapping PCRs for constructing hPg [D^413^N], hPg [D^518^N], hPg [D^139,413,518^N], and hPg [K^708^A].

Endotoxin-free preparations of the DNA constructs were used to transfect *Drosophila S2* cells using TransIT-Insect Transfection Reagent (Mirus) with EX-CELL 420 serum-free medium (Sigma) supplemented with 10% fetal bovine serum (FBS). Cells that underwent successful transfection were selected using 10–15 μg/mL puromycin. Following selection and adaptation of the S2 cells to FBS-free medium, expression of the hPg variants was carried out in spinner flasks starting with ∼4.0 × 10^6^ S2 cells in 200 mL of EX-CELL 420/0.1% pluronic acid/29250–39780 KIU aprotinin. Once the cell density reached ∼20 × 10^6^, the culture volume was increased to 1 L, and the cells were allowed to grow to a density between 20–24 × 10^6^ before the induction of hPg expression with 600 μM CuSO_4_. The culture was harvested 72–96 h post-induction. The supernatants, which contained the expressed proteins, were loaded onto individual lys-sepharose columns and purified as described by Deutsch and Mertz (1970). The purity of the hPg variants was ascertained by polyacrylamide gel electrophoresis, and molecular weights were estimated by the same method.

#### PAM and SK

The construction, expression, and purification of recombinant M-protein (PAM) and recombinant SK have been previously reported (Qiu et al., 2019; Zhang et al., 2012).

### Analytical ultracentrifugation

Sedimentation coefficients (S^0^_20,w_) of the hPg variants were determined by sedimentation velocity using the absorption optics (A_280nm_) of an analytical ultracentrifuge (Beckman Optima XL-I). The sample channels of three 2-channel centerpieces were loaded with 400 µL hPg variant at A_280nm_ values of 0.175 (0.1 mg/mL), 0.35 (0.2 mg/mL), and 0.7 (0.4 mg/L), and the reference channels were filled with 420 μL sample buffer (50 mM sodium phosphate/100 mM NaCl, pH 7.4). The samples were subjected to high-speed centrifugation at 40,000 rpm, at 20°C, with a total of 250 scans recorded at A_280nm_ every 3 min for ∼13 h. The data were analyzed by fitting into the continuous c(s) distribution model in Sedfit (Brown and Schuck, 2006). Protein and buffer parameters, which include partial specific volume, buffer density, and viscosity, were calculated from protein sequence and buffer composition using Sednterp (http://www.rasmb.org/sednterp). The effect of the lysine analog, ε-amino caproic acid (EACA), on S^0^_20,w_ of the hPg variants was similarly determined, except that the sample buffer contained, in addition to other components, 100 mM EACA.

### Plasminogen activation

Activation of hPg to hPm was measured by coupling hPm formation to the hydrolysis of H-D-Val-l-Leu-l-Lys-p-nitroanilide (S2251; Chromogenix) in 10 mM Na-Hepes/150 mM NaCl, pH 7.4, at 25°C in a thermostated spectrophotometer. A typical assay mixture in the well of a 96-well microtiter plate contained 0.2 μM hPg, 0 or 0.25 μM PAM, and 0.25 mM S2251. SK (5 nM), human uPA (2.5 nM), or human tPA (5 nM) was finally added to accelerate the reaction. The hydrolysis of S2251 by hPm to generate p-nitroaniline was continuously monitored for 2 h at A_405nm_. The slope of the linear portion of the plot of A_405nm_ vs*.* t^2^ (min^2^) was obtained using GraphPad Prism 9.0, and the initial velocity of hPm formation was calculated as described by Chibber et al. (1985).

### Surface plasmon resonance

Surface plasmon resonance (SPR) experiments were conducted in 10 mM Na-Hepes/150 mM NaCl, 3 mM EDTA/0.05% polysorbate-20, and pH 7.4 (HBS-EP+) running buffer at 25°C in a Biacore X100 (Cytiva). To determine the association (k_on_) and dissociation constants (k_off_) for the binding of the hPg variants to PAM, PAM was amine-coupled to the flow cell-2 (FC2) of a carboxymethyl dextran CM-5 chip to a response unit of 250 RU. Flow cell-1 (FC1) was treated as a blank, as described by Ayinuola et al. (2021). WT-hPg or its variants were diluted into HPS-EP+, at concentrations of 0.175–20 nM, and up to 800 nM of hPg [D^219^N] was injected into both FC1 and FC2 at a flow rate of 30 μL/s in a multicycle kinetic experiment with association and dissociation time set to 180 s and 300 s, respectively. The chip surface was regenerated between cycles by injecting 10 mM glycine-HCl, with pH 1.5. Signals from FC-1 were subtracted from those of FC-2, and the sensorgrams generated were analyzed by a 1:1 Langmuir binding model in BIAevaluation software version 3.0. The dissociation constant’s (K_D_) standard deviations were calculated from the experimental means of three independent experiments.

For the binding of hPg variants to streptokinase (SK), each hPg variant was immobilized by amine coupling on a CM-5 chip to a final response unit of ∼1200 RU, as described for PAM. Multicycle kinetic assays to determine the k_on_ and k_off_ values of these interactions were performed at SK concentrations ranging from 0.625 to 100 nM using HBS-EP+/50 μM NPGB (p-nitrophenyl-p’-guanidinobenzoate) as the analyte buffer. Association and dissociation times were 90 s and 120 s, respectively. Regeneration of the chip surface between cycles, and the data analysis, was performed as described for PAM.

### Statistical analyses

Statistical analyses were performed by one-way analysis of variance (ANOVA) with Dunnett T3 multiple comparisons test, using GraphPad Prism 9.0. Probability values (*p*-values) considered significant were set at *p* < 0.05.

## Results

### Homogeneity and molecular weight of hPg variants

Four LBS variants, including hPg [D^139^N], hPg [D^413^N], hPg [D^518^N], and hPg [D^139,413,518^N], were used in this study to investigate the importance of lysine-binding kringle domains, in intact hPg, to the tight binding of hPg to PAM. In the first three variants, critical aspartic acid residues of K1_hPg_, K4_hPg_, and K5_hPg_ were individually replaced by Asn, and all three were replaced in the last variant. We have previously shown that the replacement of Asp^219^ of K2_hPg_ to provide hPg [D^219^N] eliminates the PAM/hPg interaction (Ayinuola et al., 2021). However, this mutation does not allow us to demonstrate whether the interaction between hPg and PAM is enhanced or stabilized by LBS of K1_hPg_, K4_hPg_, and/or K5_hPg_. Moreover, a fifth hPg variant, hPg [K^708^A], is of interest to examine the significance of the salt bridge formed between Asp^219^ of K2_hPg_ and Lys^708^ of the SP domain in the conformation and activation of hPg. Thus, seven hPg variants, including WT-hPg and hPg [D^219^N] (Ayinuola et al., 2021), were used in this study.

**FIGURE 1:**
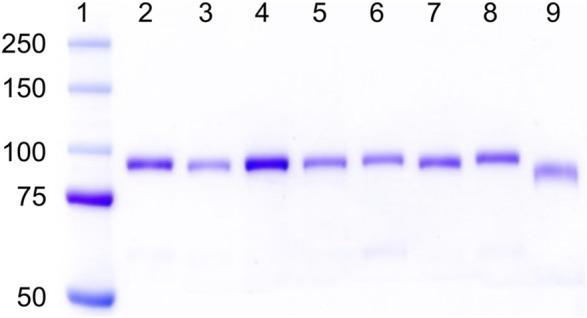
SDS-gel electrophoretograms of hPg variants on 8% Tris-glycine gel. Lane 1, pre-stained bands of protein ladder (Kaleidoscope, Bio-Rad); Lanes 2–9 show Coomassie-stained bands of hPg variants in the order of **2**, WT-Glu^1^-hPg; **3**, hPg [D^139^N]; **4**, hPg [D^219^N]; **5**, hPg [D^413^N]; **6**, hPg [D^518^N]; **7**, hPg [D^139,413,518^N]; **8**, hPg [K^708^A]; and **9**, Lys^78^-hPg.

As shown in Figure 1, all the hPg variants were purified to apparent homogeneity with yields between 25 and 55 mg/L of culture media, except for hPg [D^139,413,518^N], which gave a purified yield of ∼5 mg/L, primarily because of its poor affinity to sepharose-lysine. The low yield of this variant confirms that all the high-affinity LBS have been inactivated as expected. As shown in lanes 1 and 9 of Figure 1, Lys^78^-hPg exists at a molecular weight below that of WT-Glu^1^hPg due to the absence of the AP domain. Thus, it is evident that all of the LBS variants, including hPg [K^708^A], which migrates with relative mobility close to that of WT-hPg, exist in their Glu^1^-hPg form of ∼92 kDa (Ayinuola et al., 2021).

### Replacement of non-LBS Lys^708^ relaxes the conformation of Glu^1^-hPg

Since LBS regulates the conformation of hPg, all constructed variants were subjected to conformational analysis by the determination of sedimentation coefficient value, S^o^_20,w_, a parameter dramatically reduced when hPg transitions from a tight closed (T) to a loose open (L) conformation (Brockway and Castellino, 1972). The results of the S^o^_20,w_ analyses are summarized in Table 2. As shown from the results obtained without EACA, WT-hPg and hPg [D^139^N] have identical S^o^_20,w_ values of ∼5.4 S, indicating that K1_hPg_ may not play a major role in regulating the conformation of hPg. Other LBS variants sedimented more slowly with a reduction of ∼0.4–0.7 units in their S^o^_20,w_ values. The triple variant carrying a combined mutation of LBS residues in K1_hPg_, K4_hPg_, and K5_hPg_ has the lowest S^o^_20,w_ value, implying that it is in a more relaxed conformation than any other variants. This is followed by hPg [D^219^N]. Of all the LBS variants, the replacement of Asp^518^ of the LBS of K5_hPg_ resulted in the lowest degree of change in S^o^_20,w_.

**TABLE 2:** S^o^_20,w_ values of the hPg variants.

**Plasminogen variant**	**(S** ^**o**^_**20,w**_ **)** ^a^	**(S** ^**o**^_**20,w**_ **)** ^b^
WT-hPg	5.41 ± 0.03	4.49 ± 0.01_**_
hPg [D^139^N]	5.39 ± 0.02	4.50 ± 0.01_****_
hPg [D^219^N]	4.83 ± 0.03****	4.47 ± 0.01_*_
hPg [D^413^N]	4.96 ± 0.01***	4.48 ± 0.04_*_
hPg [D^518^N]	5.06 ± 0.04**	4.53 ± 0.02_**_
hPg [D^139,413,518^N]	4.75 ± 0.03****	4.50 ± 0.01_*_
hPg [K^708^A]	5.11 ± 0.03*	4.49 ± 0.01_**_

^a^ Experiments were performed in 50 mM Na-phosphate/100 mM NaCl. The asterisks indicate probability (p) values obtained for pairwise comparison between WT-hPg and the other variants. *p < 0.05; **p < 0.01; ****p < 0.001.

^b^ Experiments performed in 50 mM Ma-phosphate/100 mM NaCl/100 mM EACA. Asterisks shown as superscripts indicate probability (p) values obtained for pairwise comparison between WT-hPg and the order variants. In contrast, those shown as subscripts compare the S_20,w_ of the same hPg variant in a buffer lacking EACA with those obtained in a buffer with EACA.

Interestingly, the S^o^_20,w_ of hPg [K^708^A] is ∼0.3 unit lower than that of WT-hPg, demonstrating that this variant exists in a slightly more open conformation than Glu^1^-hPg. This suggests that the interaction between Lys^708^ and Asp^219^ modulates the hPg conformation. In the buffer containing EACA, all the hPg variants transitioned from either the closed conformation, as in hPg WT and hPg [D^139^N]; slightly relaxed as in hPgD^518^N and hPgK^708^A; or more relaxed as in hPgD^219^N, hPgD^413^N, and hPg D^139,413,518^N, to the fully relaxed and open conformation of ∼4.5 S, consistent with ∼1.0 S unit change characteristics of the transition of hPg from a closed to an open form (Brockway and Castellino, 1972).

### Loss of Asp^413^ and Lys^708^ fuels rapid association of hPg and PAM

**FIGURE 2:**
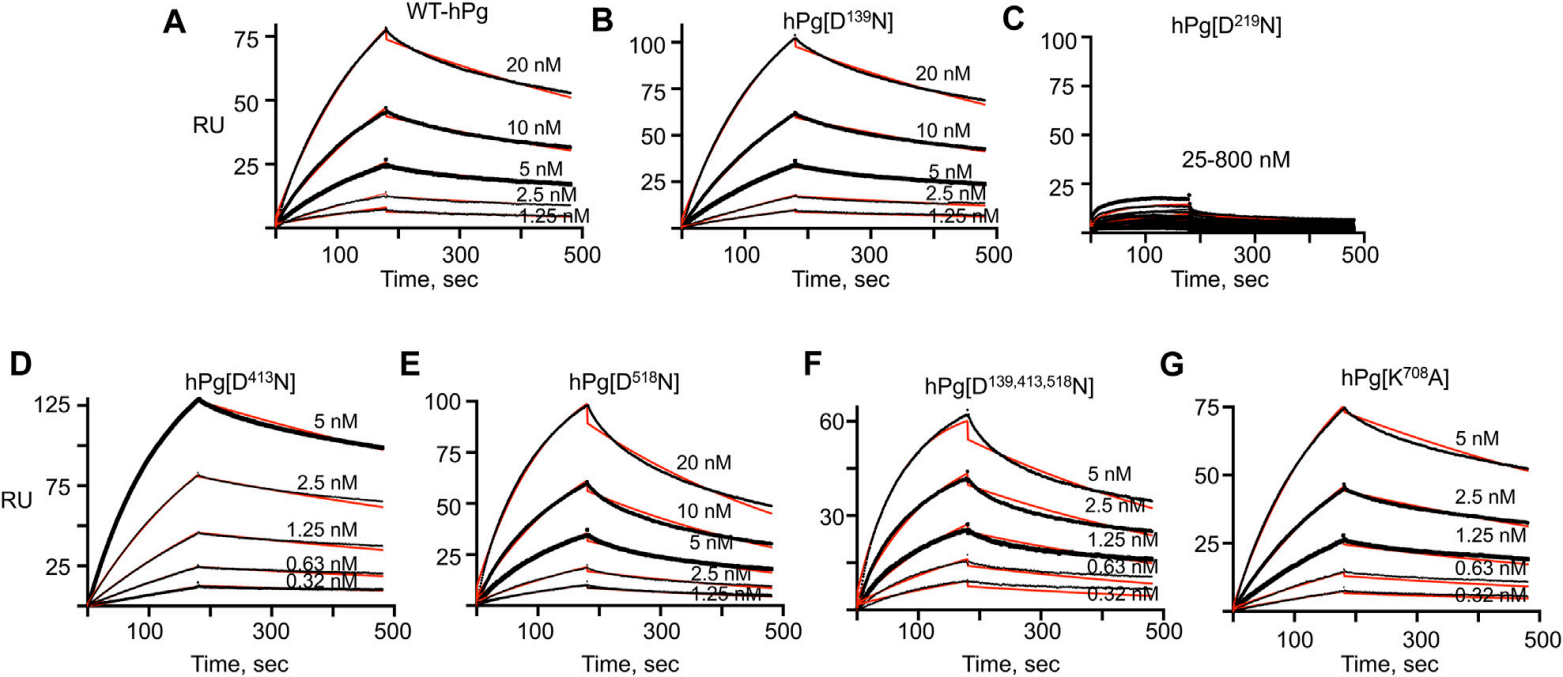
SPR sensorgrams of hPg variants’ interaction with PAM. PAM was amine-coupled to a CM-5 chip to a target level of 250 RU, and each hPg variant was injected on the chip in a multicycle kinetic experiment at the indicated concentrations. The black lines show the measured association (0–180 s) and dissociation curves (181–480 s) for the interaction of the hPg variants and PAM. The red lines show the best fit for the association and dissociation rate constants of the hPg variants to PAM using a 1:1 Langmuir model. **(A)** WT-hPg, **(B)** hPg[D^139^N], **(C)** hPg[D^219^N], **(D)** hPg[D^413^N], **(E)** hPg[D^518^N], **(F)** hPg[D^139, 413, 518^N] and **(G)** hPg[K^708^A].

The association and dissociation curves for the binding of the hPg variants to immobilized PAM are given by the sensorgrams in Figure 2. Analyses of the sensorgrams showed that hPg variants hPg [D^413^N] and hPg [D^139,413,518^N] have ∼10X higher affinities for PAM compared to WT-hPg (Table 3). This is due to a fast on-rate upon the replacement of Asp^413^ of K4_hPg_. In addition the association rate of hPg [K^708^A] is 5X that of WT-hPg, leading to a ∼4-fold increase in its affinity for PAM. The replacement of LBS residues of K1_hPg_ and K5_hPg_ does not significantly alter the binding kinetics of hPg to PAM. Overall, the dissociation rates of the variants are about the same. These similar off-rates indicate that the altered LBS residues are not involved in stabilizing the hPg/PAM interaction.

**TABLE 3:** Binding constants of hPg variants/PAM as determined by SPR.

**Plasminogen variant**	**K**_**on**_ **(1/Ms) x 10** ^**4**^	**K**_**off**_ **(1/s) x 10** ^**−**^ **^4^**	**K**_**D (nM)**_
WT-hPg	24 ± 1	13 ± 1	5.4 ± 1.1
hPg [D^139^N]	41 ± 8^ns^	16 ± 1^ns^	4.0 ± 0.6^ns^
hPg [D^219^N]	ND^a^	ND^a^	ND^a^
hPg [D^413^N]	134 ± 4****	9.4 ± 0.1*	0.7 ± 0.1*
hPg [D^518^N]	36 ± 3^ns^	24 ± 1****	6.7 ± 0.3^ns^
hPg [D^139,413,518^N]	251 ± 30*	17 ± 2*	0.7 ± 0.1*
hPg [K^708^A]	97 ± 4****	12 ± 1^ns^	1.2 ± 0.1*

Asterisks indicate probability (p) values obtained for pairwise comparison between K_on_, K_off_, and K_D_ of WT-hPg and the other variants. *p < 0.05; ****p < 0.0001; ns, not significant. ND, too weak to be determined.

### The LBS of hPg participates in functions other than maintenance of slowly activatable hPg conformation

#### Activation of hPg with SK

**FIGURE 3:**
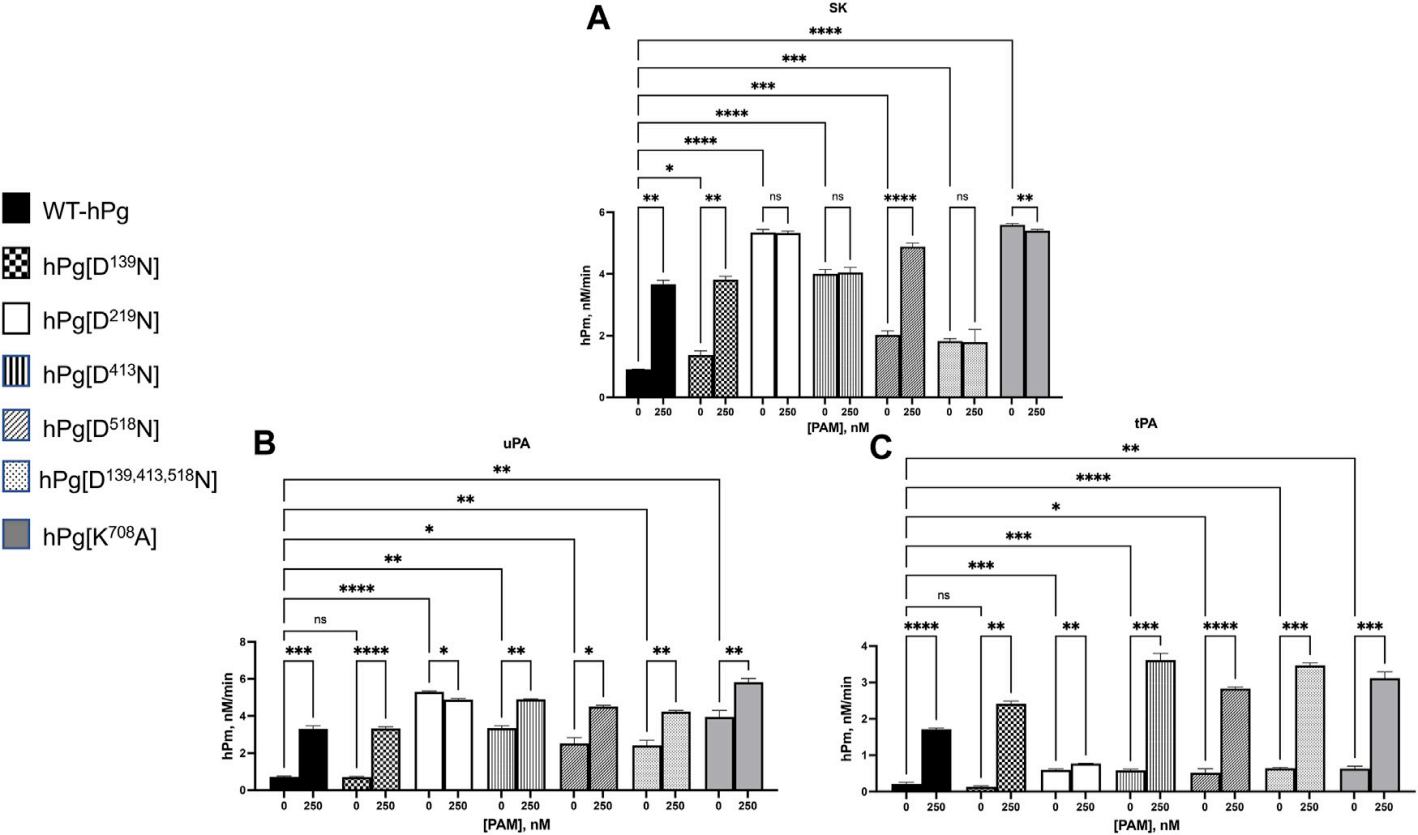
Activation of hPg variants by plasminogen activators at 25°C. Activation of hPg was coupled to the cleavage of chromogenic substrate S2251 (H-D-Val-L-Leu-L-Lys-p-nitroanilide) in 50 mM Na^+^-Hepes/100 mM NaCl, pH 7.4. Each assay mixture contained 200 nM hPg variant and 0.25 mM S2251, 0 or 250 nM PAM, and **(A)** 5 nM SK as the hPg activator; **(B)** 2.5 nM uPA as the hPg activator; and **(C)** 5 nM tPA as the hPg activator. The generation of p-nitroaniline from S2251 by the generated hPm was continuously measured at A_405nm_ for 90 min. The rate function of hPg activation was calculated as the slope of the linear portions of A_405nm_ vs*.* t^2^. Probability values obtained from pairwise comparisons between WT-hPg and the other variants at 0 nM and between each hPg variant at 0 and 250 nM PAM are shown. Here, * is *p* < 0.05, ** is *p* < 0.01, *** is *p* < 0.001, **** is *p* < 0.0001, and ns is not significant.

To expand the binding data obtained for the hPg/PAM interactions, we examined the stimulatory effect of PAM binding to hPg on the SK-mediated activation of the hPg variants. Consistent with the conformational differences observed between the variants, WT-hPg and hPg [D^139^N], both of which exist in the fully closed conformation, showed the slowest activation rates in an assay without PAM (Figure 3A). Upon the addition of PAM, the activation rates of these two variants were enhanced ∼4-fold. hPg [D^518^N] has a slightly relaxed conformation, which agrees with its somewhat faster activation rate than WT-hPg. As expected, based on the binding result of this variant with PAM, the addition of PAM generated more hPm per unit time. Three variants, hPg [D^219^N], hPg [D^413^N], and hPg [D^139,413,518^N], exist in the more relaxed conformation. Of these three variants, hPg [D^219^N] and hPg [D^413^N] showed the optimal activation rate possible with SK, and the addition of PAM did not increase their rates of activation. However, unexpectedly, the rate of hPm generation from hPg [D^139,413,518^N], although faster than that of WT-hPg, is inconsistent with its more relaxed conformation. Moreover, despite its tight binding to PAM, its activation rate is not responsive to the presence of PAM. Surprisingly, although hPg [K^708^A] shows a slightly relaxed conformation, it is optimally activatable, and PAM does not further stimulate its activation by SK. Overall, the activation profile of the hPg variants recapitulates the mechanism by which PAM enhances the rate of hPg activation in that PAM produces a conformational switch upon binding to hPg. hPg [D^219^N], hPg [D^413^N], and hPg [K^708^A] are already in the optimally activatable conformations required by SK, plausibly due to the activation loop that harbors the R^561^-V^562^ activation site being readily exposed in these conformations. Hence, the lack of binding of PAM to hPg [D^219^N] or its binding to hPg [D^413^N] and hPg [K^708^A] may not necessarily overcome additional conformational barriers to activating hPg.

#### Activation by hPg activators, uPA and tPA

While it is clear from the AUC data, the SPR- and SK-mediated activation results show that individual mutations of LBS residues of K1_hPg_, K4_hPg_, and K5_hPg_ do not negatively impact the hPg/PAM interaction. However, it is not clear why a combination of these mutations is less favorable to SK-mediated activation of hPg [D^139,413,518^N]. To better characterize this variant, we evaluated the activation rates of all the hPg variants using uPA and tPA as activators. Human uPA and tPA serve as valuable tools in this instance because they are serine proteases, and, unlike SK, they directly cleave the scissile R^561^-V^562^ bond in the activation loop of hPg. On the other hand, SK has no intrinsic proteolytic activity but functions similarly to a coenzyme forming a complex with hPg and generates protease activity. The SK-hPg complex, in turn, activates free hPg. Thus, mutations that diminish initial complex formation will hinder hPg activation.

The results for the uPA activation for WT-hPg, hPg [D^139^N], hPg [D^219^N], and hPg [D^518^N] (Figure 3B) followed the same trend observed using SK as the activator. However, the activation rates of hPg [D^413^N] and hPg [K^708^A], while still rapid, unlike the SK-mediated reactions, were further enhanced by the addition of PAM. Moreover, the rate of activation of hPg [D^139,413,518^N] is only slightly lower than those of hPg [D^413^N] and hPg [K^708^A] and is stimulated by PAM.

For tPA as an activator, Figure 3C shows that in assay mixtures devoid of PAM, tPA does not discriminate between a slight or a more relaxed hPg conformation. A small alteration that slightly opens the hPg molecule places hPg [D^219^N], hPg [D^413^N], hPg [D^518^N], hPg [D^139,413,518^N], and hPg [K^708^A] at the same activation potential with activation rates ∼3x faster than those of WT-hPg and hPg [D^139^N]. Upon the addition of PAM, the activation rates of all the hPg variants were further enhanced, except for hPg [D^219^N].

Largely, the activation of the hPg variants suggests that the replacement of the LBS residue of K5_hPg_, or the simultaneous replacement of LBS residues of K1_hPg_, K4_hPg_, and K5_hPg_, inhibits complex formation between SK and hPg. Moreover, the activation profiles highlight the difference in the mechanism of SK, uPA, and tPA activation.

**FIGURE 4:**
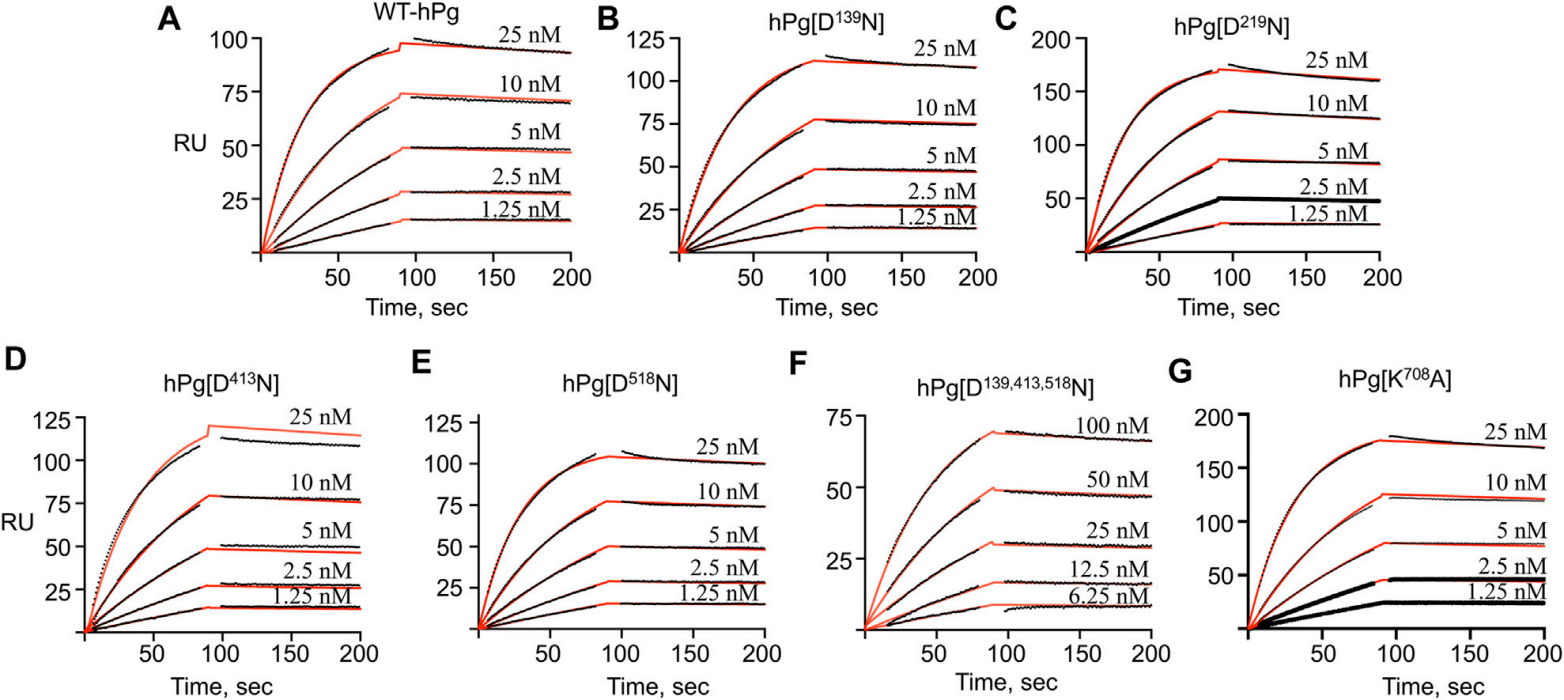
SPR sensorgrams for the binding of hPg variants to SK. hPg and hPg variants were amine-coupled to a CM-5 chip, and SK was injected on each chip at the indicated concentrations. The black lines showed the measured association (0–90 s) and dissociation (91–210 s) curves of the SK interaction with hPg. p-NPGB was added to prevent activation of the hPg bound to SK. Association and dissociation rate constants were obtained by fitting the data using a 1:1 Langmuir model, as shown by the red lines. **(A)** WT-hPg, **(B)** hPg[N^139^D], **(C)** hPg[D^219^N], **(D)** hPg[D^413^N], **(E)** hPg[D^518^N], **(F)** hPg[D^139, 413, 518^N] and **(G)** hPg[K^708^A].

To gain further insight into the reasons for the less favorable activation of hPg [D^139,413,518^N] compared to its other conformationally more relaxed counterparts, we determined the dissociation constants, K_D_, for the binding of the hPg variants to SK by SPR. The average K_D_ value for the interaction is ∼0.3 nM for all the variants except for hPg [D^139,413,518^N], the K_D_ of which 2.1 nM (Figure 4; Table 4), reflecting a ∼7X slower association rate with SK. Notably, none of the mutations created in the hPg variants altered the k_off_. Herein, it is evident that a key reason for the retarded activation of hPg D^139,413,518^N is the slow rate of forming the initial activator complex.

**TABLE 4:** Binding constants of hPg variants to SK as determined by SPR.

**hPg variant**	**K**_**on**_ **(1/Ms) x 10** ^**4**^	**K**_**off**_ **(1/s) x 10** ^**−**^ **^4^**	**K**_**D (nM)**_
WT-hPg	139 ± 8.4	4.05 ± 0.7	0.29 ± 0.04
hPg [D^139^N]	113 ± 3.0^ns^	3.02 ± 0.33^ns^	0.28 ± 0.02^ns^
hPg [D^219^N]	155 ± 12.9^ns^	4.66 ± 1.01^ns^	0.3 ± 0.04^ns^
hPg [D^413^N]	122 ± 20.9^ns^	3.02 ± 1.13^ns^	0.26 ± 0.14^ns^
hPg [D^518^N]	129 ± 13.3^ns^	3.4 ± 0.58^ns^	0.27 ± 0.02^ns^
hPg [D^139,413,518^N]	20.6 ± 0.5***	4.49 ± 0.74^ns^	2.1 ± 0.3***
hPg [K^708^A]	136 ± 14.4^ns^	2.82 ± 0.57^ns^	0.21 ± 0.01^ns^

Asterisks indicate probability (p) values obtained for pairwise comparison between K_on_, K_off_, and K_D_ of WT-hPg and the other variants. ***p < 0.001; ns, not significant.

## Discussion

The binding of hPg to cellular receptors plays a vital role in modulating hPg activation, chiefly due to conformational changes in hPg that accompany the binding events. Such conformational alterations are induced by competitive binding of the C-terminal lysine or lysine isosteres to the LBS of hPg, displacing the intramolecular interactions offered by the LBS that keep hPg in a tight activation-resistant conformation. Many pathogenic bacteria employ surface-associated hPg receptors to immobilize hPg on the cell surface. The multifunctional M-protein of group A *S. pyogenes* (GAS) functions in this capacity to indirectly or directly capture hPg on the GAS surface. Indirect hPg binding is achieved through initial high-affinity binding to fibrinogen, which in turn binds hPg (Herwald et al., 2004; Walker et al., 2005; Smeesters et al., 2010a; Smeesters et al., 2010b; Qiu et al., 2018). Direct hPg binding is mediated by the subclass of M-proteins called PAM, expressed only in pattern D skin-trophic GAS strains (Berge and Sjobring, 1993; Wistedt et al., 1995). PAM binds hPg with the aid of lysine isosteres, interacting with critical residues of K2_hPg_ LBS (Rios-Steiner et al., 2001; Yuan et al., 2019). Binding experiments using individual kringle domains showed that K1_hPg_, K4_hPg_, and K5_hPg_ do not participate in PAM/hPg interaction (Wistedt et al., 1998). In the current study, we explore the contributions of these kringle domains to hPg binding in the context of an intact hPg molecule. Our present study showed that hPg variants lacking critical aspartate residues of the anionic loci of K1_hPg_, K4_hPg_, and K5_hPg_ bind tightly to PAM with very low nanomolar K_D_ values, reiterating the non-involvement of these LBS in hPg binding by PAM. However, the observation that the replacements of Asp^413^, Lys^708^, and a combination of Asp^413^ and Asp^518^ produced conformational changes that allow rapid association of PAM and hPg suggests, on one hand, that the opening of the closed hPg conformation exposes residue exosites of K2_hPg_ that facilitates the interaction. On the other hand, the data suggest that steric hindrance to K2_hPg_ exists in the closed hPg conformation and that K2_hPg_ is readily exposed for rapid PAM interaction in the opened hPg conformation.

In line with conformational perturbations that ensue following the interaction of hPg LBS with receptors, we demonstrated that the replacement of critical aspartate residues in homologous locations of K1_hPg_, K4_hPg_, and K5_hPg_, similar to our previous report on hPg [D^219^N] (K2_hPg_ variant), deprived hPg of the critical anionic residue required to maintain its closed conformation in hPg [D^413^N] (K4_hPg_ variant) and hPg [D^518^N] (K5_hPg_ variant). However, hPg [D^139^N] (K1_hPg_ variant) maintained a tight activation-resistant conformation regardless of the mutation introduced. These findings are consistent with the X-ray crystal structural study of hPg in which only K1_hPg_ was shown to be positioned away from the hPg core, having no interaction with the AP or SP domain (Law et al., 2012). The implication is that although K1_hPg_ has the highest lysine-binding affinity, it does not contribute to conformational changes that occur in hPg. Thus, receptors that strictly depend on K1_hPg_ for interaction with hPg would not offer any stimulatory effect to hPg activation. This argument aligns with the reports that saturation of the high-affinity binding site of hPg by EACA or tranexamic acid does not induce a significant conformational change in hPg (Markus et al., 1978a; Markus et al., 1978b; Markus et al., 1979). An appreciable degree of conformational change was observed in hPg [D^518^N]. However, this is a slight shift in the conformation of hPg compared to the changes observed in hPg [D^219^N] and hPg [D^413^N]. Furthermore, the conformational change in hPg [D^518^N] only led to a 2X increase in hPg activation potential by SK, unlike a 4–5-fold increase observed upon the addition of PAM and in hPg [D^219^N] and hPg [D^413^N]. K5_hPg_ has been shown to interact solely with the AP domain of hPg (Law et al., 2012). Moreover, a biophysical study indicated that benzamidine, a ligand that binds only at K5_hPg_ LBS but not at K1_hPg_–K4_hPg_, does not induce the formation of the fully extended hPg conformation (Marshall et al., 1994). Consistent with these reports, our current findings confirm that K5_hPg_ LBS is not involved in an intramolecular interaction that causes a global conformational change within hPg upon its removal. This signifies that the conformational change in hPg [D^518^N] does not influence the SP domain in a manner that significantly exposes the activation loop of hPg. However, the conformational impediments afforded by the interaction between the non-K5_hPg_ LBS and SP domain to the activation of hPg [D^518^N] were overcome when PAM was added to its SK-catalyzed activation reaction mixture. This behavior of hPg [D^518^N] demonstrates why hPg [D^219^N], hPgD^413^N, and hP [gK^708^A] are readily activatable by SK, as explained below.

In the crystal structure of hPg, other than the interaction of Asp^413^ of K4_hPg_ with Arg^68^ and Lys^70^ of the AP domain, an interface formed between K4_hPg_, the activation loop, and the SP domain positioned the inter-kringle loop of K3_hPg_ and K4_hPg_ in a manner that blocked the activation sequence (Law et al., 2012). Thus, the replacement of Asp^413^, as observed in hPg [D^413^N], will eliminate its interaction with the AP domain and plausibly alter the position of K3_hPg_/K4_hPg_ inter-kringle loop, leading to rapid activation of hPg. In the case of K2_hPg_, two loops in the SP domain extensively interact with K2_hPg_. Moreover, Cl^−^ coordinates multiple interactions between K2_hPg_ and SP domain, and a salt bridge exists between Lys^708^ of the SP domain and Asp^219^/Glu^221^ of K2_hPg_ LBS (Urano et al., 1987b; Law et al., 2012). These properties of K2_hPg_, highlighted by the crystal structure, agree with our findings that of all the replacements involving a single LBS residue, the substitution of Asp^219^ generated the largest conformational change that accelerated hPg activation. Thus, the elimination of the K2_hPg_/SP domain interaction appears to be the most critical event that optimally exposes the Arg^561^-Val^562^ activation site of hPg. This is especially evident in hPg [K^708^A], a variant exhibiting a minimal conformational change but optimally activatable by all hPg activators. First, the results of hPg [K^708^A] indicate that the extent of conformational change, even though necessary for rapid hPg activation, is not as significant as a change that exposes the activation loop. Second, it suggests that interactions between K2_hPg_ and the SP domain are the most critical interactions that regulate hPg activation, and PAM-binding disrupts these interactions. Finally, the association rate for the binding of PAM to the hPg variants combined with the activation of hPg by SK suggests that the position of K2_hPg_ is likely altered in hPg [D^219^N], hPg [D^413^N], and hPg [K^708^A], such as to expose the activation loop. Consequently, the binding of PAM to these variants will be redundant for the rate enhancement of hPg activation. A significant increase in the activation rates of these hPg variants observed upon the addition of PAM to their tPA-catalyzed reactions suggests that the presence of intact K2_hPg_ LBS in hPg [D^413^N] and hPg [K^708^A] enabled hPg-PAM complex formation and, by extension, ternary complexes of hPg-PAM-tPA, which alters the catalysis by tPA.

Of all the hPg variants used in this study, hPg [D^139,413,518^N] exhibits the largest shift in S^o^_20,W_ value, implying that it exists in the most relaxed conformation. It would be expected that the activation rate of this variant will be similar to that of hPg [D^413^N]. However, the activation rate is indeed slower. A simple explanation for this is that the activation rate of hPg [D^139,413,518^N] reflects an interplay between an open conformation of hPg, wherein the activation loop is exposed, and the loss of essential residues of the LBS. Thus, even though the conformation is relaxed, for SK, the initial event of rapid complex formation is affected by the absence of important LBS residues. This is clearly seen in the SPR binding data of this variant with SK. In addition, we found that, unlike uPA- and tPA-catalyzed reactions, the lysine analog, EACA, would not stimulate the activation of hPg by SK. However, if a preformed complex of SK-hPg is used as the activator, EACA does stimulate the activation. This is consistent with earlier reports that EACA potently inhibits the binding of SK to hPg (Conejero-Lara et al., 1998; Lin et al., 2000), thereby strengthening the point that the slow activation rate of hPg [D^139,413,518^N] by SK is due to the inactivation of high-affinity LBS. The modulatory effect of the kringle domains of hPg on the interaction between hPg and SK was previously demonstrated by a binding saturation experiment, which revealed that both Glu^1^-hPg and mini-hPg (hPg devoid of K1_hPg_—K4_hPg_) possess equal binding affinity of ∼0.5 nM for SK, a value that is two orders of magnitude lower than that obtained with micro-hPg (hPg devoid of all five kringle domains). Therefore, it was suggested that K5_hPg_ is the only kringle domain required for low nanomolar high-affinity interaction of hPg and SK (Lin et al., 2000). However, our current data showed that inactivation of either K1_hPg_, K4_hPg_, or K5_hPg_ through the individual replacement of Asp^139^, Asp^413^, or Asp^518^ did not affect the interaction between hPg and SK, suggesting that in the absence of one high-affinity LBS, another can function to enable rapid interaction of SK and hPg. Based on a K_D_ of 2.1 nM obtained for the interaction of SK and hPg [D^139,413,518^N], the activation mixture should contain ∼70% activator complex, albeit the observed rate is slower than expected for a 70% complex. Perhaps the replaced LBS residues are also involved in the interaction of the activator complex with the hPg substrate, consistent with enzyme kinetics studies which showed that lysine, EACA, and tranexamic acid are competitive inhibitors of hPg activation by uPA (Peltz et al., 1982; Wu et al., 2019). Hence, apart from its importance in the formation of SK/hPg activator complex, the LBSs seem to play an important role in the formation of enzyme-substrate complexes between plasminogen activators and hPg.

In summary, we show in the current study that K1_hPg_, K4_hPg_, and K5_hPg_ do not directly influence the high-affinity interaction of full-length hPg with PAM, but conformational perturbations that relax the hPg molecule facilitate rapid binding with PAM. Inactivation of the LBS of K1_hPg_ does not alter hPg conformation, and the loss of the K5_hPg_ LBS does not induce a global conformational change in hPg. Substitution of critical K4_hPg_ and K2_hPg_ LBS residues places hPg at optimal activation potential, rapidly activatable by all hPg activators. However, the single replacement of Asp^219^ of K2_hPg_ not only resulted in the largest conformational change of all the inactivated LBS, but the substitution of Lys^708^, a binding partner of Asp^219^, sufficiently rendered hPg optimally activatable. Thus, the order of increasing influence of LBS of the hPg kringle domains on its conformation and activatability is K1_hPg_ < K5_hPg_ < K4_hPg_ < K2_hPg_. In conclusion, beyond a more relaxed extended hPg conformation, exposure of the activation loop, alongside the presence of appropriate LBS residues, is a prerequisite for rapid conversion of hPg to hPm by plasminogen activators**.**

## Data Availability

The original contributions presented in the study are included in the article. Further inquiries can be directed to the corresponding author.
